# Supplementary material for: Prediction of antigenic peptides of SARS- CoV-2 pathogen using machine learning
Source: PeerJ Comput Sci. 2024 Oct 10;10:e2319. doi: 10.7717/peerj-cs.2319 (PMC11623221; doi:10.7717/peerj-cs.2319)
Supplement: Supplemental Information 1 [file peerj-cs-10-2319-s001.docx]

Appendix 1 **Grid search results**

| **Hyperparameter** | **Values Searched** | **Best Value** |
| --- | --- | --- |
| Learning Rate | 0.01, 0.03, 0.05, 0.1 | 0.05 |
| Maximum Depth | 3, 5, 7 | 5 |
| Number of Trees | 50, 100, 200, 300 | 200 |
| Subsample Ratio | 0.5, 0.7, 0.8, 0.9 | 0.8 |
| Minimum Child Weight | 1, 5, 10, 20 | 10 |
