# Supplementary material for: Prediction of antigenic peptides of SARS- CoV-2 pathogen using machine learning
Source: PeerJ Comput Sci. 2024 Oct 10;10:e2319. doi: 10.7717/peerj-cs.2319 (PMC11623221; doi:10.7717/peerj-cs.2319)
Supplement: Supplemental Information 12 [file peerj-cs-10-2319-s012.docx]

Supplementary Algorithm S1 **Grid search hyperparameter tuning**

| **Algorithm 1 .** Grid search hyperparameter tuning | | | |
| --- | --- | --- | --- |
| **Start** | | | |
|  | **Input:**  Model: a machine learning model (XGBoost classifier in this study) to be trained and tested  Hyperparameters: a dictionary of hyperparameter ranges to search through  X_train, y_train: training data and labels  X_test, y_test: testing data and labels | | |
|  | **Output:**  Best hyperparameters: a dictionary containing the best hyperparameters found during the grid search  Best score: the best score achieved by the model during the grid search | | |
| **1** | Define the hyperparameters to be searched over  **hyperparameters_to_search** = Hyperparameters.keys () | | |
| **2** | Generate all possible combinations of hyperparameters  **parameter_combinations** = cartesian_product (*Hyperparameters.values ()) | | |
| **3** | Train and test the model on each combination of hyperparameters  **best_score** = None  **best_hyperparameters** = None | | |
| **4** | **for** parameters in parameter_combinations: | | |
| **5** |  | Train the model on the training data using the current hyperparameters  model.set_params(**parameters)  model.fit (X_train, y_train) | |
| **6** |  | Evaluate the model on the testing data using the current hyperparameters  **score** = model.score (X_test, y_test) | |
| **7** |  | Update the best hyperparameters and score if the current score is better | |
| **8** |  | **if** best_score is None or score > best_score: | |
| **9** |  |  | **best_score** = score  **best_hyperparameters** = parameters |
| **10** |  | **End if** | |
| **11** | **End for** | | |
| **12** | Return the best hyperparameters and score  return **best_hyperparameters**, **best_score**  Retrain the classifier on the entire dataset using the optimal combination of hyperparameters | | |
| **13** | **End** | | |
